# Dr. Ezra John Guyott

**Published:** 1914-10

**Authors:** 


Dr. Ezra John Guyott, Hahnemann of Chicago, 1882, died at his home in Utica, June 22, aged 60.

